# Pro-Survival Factor EDEM3 Confers Therapy Resistance in Prostate Cancer

**DOI:** 10.3390/ijms23158184

**Published:** 2022-07-25

**Authors:** Emma Scott, Rebecca Garnham, Kathleen Cheung, Adam Duxfield, David J. Elliott, Jennifer Munkley

**Affiliations:** 1 Centre for Cancer, Biosciences Institute, Newcastle University, Newcastle-Upon-Tyne NE1 3BZ, UK; r.garnham@newcastle.ac.uk (R.G.); a.duxfield@newcastle.ac.uk (A.D.); david.elliott@newcastle.ac.uk (D.J.E.); 2 Bioinformatic Support Unit, Newcastle University, Newcastle-Upon-Tyne NE1 3BZ, UK; kathleen.cheung@outlook.com

**Keywords:** EDEM, N-glycosylation, prostate cancer, ER-stress, radiotherapy

## Abstract

Prostate cancer is the most common cancer in men, and it is primarily driven by androgen steroid hormones. The glycosylation enzyme EDEM3 is controlled by androgen signalling and is important for prostate cancer viability. EDEM3 is a mannosidase that trims mannose from mis-folded glycoproteins, tagging them for degradation through endoplasmic reticulum-associated degradation. Here, we find that *EDEM3* is upregulated in prostate cancer, and this is linked to poorer disease-free survival. Depletion of EDEM3 from prostate cancer cells induces an ER stress transcriptomic signature, and EDEM3 overexpression is cyto-protective against ER stressors. *EDEM3* expression also positively correlates with genes involved in the unfolded protein response in prostate cancer patients, and its expression can be induced through exposure to radiation. Importantly, the overexpression of EDEM3 promotes radio-resistance in prostate cancer cells and radio-resistance can be reduced through depletion of EDEM3. Our data thus implicate increased levels of EDEM3 with a role in prostate cancer pathology and reveal a new therapeutic opportunity to sensitise prostate tumours to radiotherapy.

## 1. Introduction

Prostate cancer is the leading cause of cancer-related deaths amongst men globally, with over 200,000 new cases diagnosed in the US each year and over 34,000 prostate cancer-related deaths annually in the US [1,2]. As it stands, therapeutic options include surgery, radiotherapy, hormone targeting therapies and chemotherapeutic agents. To improve clinical management of the disease, there is a pressing need to understand the biological processes which underpin prostate cancer disease progression and response to therapeutic agents [3]. In its early stages, prostate cancer growth is dependent upon androgen receptor signalling, hence, androgen biology has been a major focus in the field to date. We recently identified a clinically relevant, androgen-regulated gene signature. In particular, glycosylation is a target for androgen control in prostate cancer [4]. Glycosylation is the enzymatic addition of glycans to target substrates, and aberrant patterns of glycosylation have been identified in multiple human malignancies [5,6,7,8,9]. Changes to the glycome (the complete pattern of glycan modifications present on a cell or tissue) have recently been shown to be an important feature of prostate carcinogenesis, and enzymes which control these changes are exciting new therapeutic targets and biomarkers for the disease [10,11,12,13,14,15,16,17,18].

Androgens regulate glycosylation in prostate cancer by regulating the gene expression of enzymes responsible for building and modifying glycans [4,12]. In our previous work, we identified *ER Degradation Enhancing Alpha-Mannosidase Like Protein 3 (EDEM3)* as an androgen-regulated gene in prostate cancer [4]. *EDEM3* encodes a member of the glycosyl hydrolase family of proteins, and is one of three EDEM paralogs (alongside *EDEM1* and *EDEM2*) [19,20,21]. The EDEM proteins are key members of the endoplasmic reticulum-associated degradation pathway (ERAD), a process responsible for degradation of mis-folded proteins as a mechanism to protect the cell from ER stress [21,22]. This process, if activated by the accumulation of mis-folded proteins in the ER, promotes the unfolded protein response (UPR) [23]. Triggering of the UPR stimulates three key signalling pathways, mediated by the three stress sensors ATF6, PERK and IRE-1 [24]. The UPR is an adaptive response, often observed in cancer cells, in response to ER stress through various causes, such as hypoxia, DNA damage and metabolic strain. The purpose of the UPR is to protect the cell; however, when hyper-activated it can lead to cell death. Therapies such as chemotherapeutic agents and radiotherapy induce ER stress, resulting in cancer cell death and recently, proteins involved in the ER stress response have become attractive therapeutic targets to sensitise cancer patients to these types of treatment [25,26,27]. Although EDEM3 has been linked to cell survival, how it does this has not been well understood. To understand the importance of EDEM genes in prostate cancer, we performed transcriptomic analysis of prostate cancer cell lines and patient tissue. This established *EDEM3* as the clinically significant EDEM paralog upregulated in prostate tumours and revealed *EDEM3* expression is associated with activity of the UPR pathway. Using in vitro ER stress and radiosensitivity assays, we show EDEM3 as a pro-survival factor that is preferentially upregulated as a protective measure in response to ER stress. Consistent with this, EDEM3 is induced following exposure to radiation, and high levels of EDEM3 can confer radio-resistance to cancer cells (an effect which can be reversed by targeting EDEM3). Our findings implicate increased levels of EDEM3 in prostate cancer pathology and identify a new therapeutic opportunity to sensitise prostate tumours to radiotherapy.

## 2. Results

### 2.1. EDEM3 Upregulation Is Associated with a Poor Disease-Free Survival in Prostate Cancer

**Figure 1:**
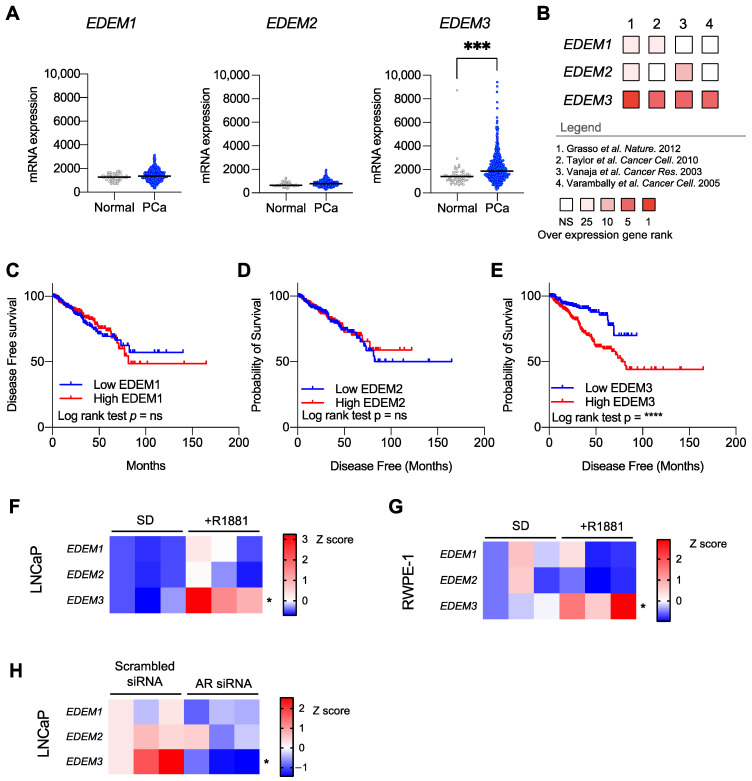
*EDEM3* upregulation is associated with a reduced disease-free survival in prostate cancer (PCa). (**A**) Analysis of *EDEM* gene expression in The Cancer Genome Atlas (TCGA) prostate adenocarcinoma cohort (n = 497) compared with normal prostate samples (52). (**B**) Meta-analysis of *EDEM1/2/3* gene expression in prostate cancer. Four independent prostate cancer gene expression data sets (n = 366) were analysed for *EDEM1/2/3* gene expression in prostate cancer patients compared with patients with normal prostates. Data shown are of overexpression gene rank in each dataset. Legend includes details of the independent cohorts. NS = not significant, 25 = top 25%, 10 = top 10%, 5 = top 5% and 1 = top 1%. Data accessed using Oncomine [29,30,31,32]. (**C**–**E**) Kaplan–Meier plot showing disease-free survival for prostate cancer patients stratified based on low (bottom 50%) or high (top 50%) *EDEM3* expression. Analysis includes 471 prostate cancer patients from TCGA PRAD cohort, accessed via CBioPortal. *p* value was calculated by log rank test. (**F**,**G**) Heatmaps showing mRNA levels of *EDEM1/2/3* in both LNCaP and RWPE-1 cultured in either steroid-depleted (SD) conditions or stimulated with 10 nM R1881 for 24 h. n = 3 and data are presented as z-scores. (**H**) Heatmap showing mRNA levels of *EDEM1/2/3* in LNCaP cells following androgen receptor siRNA knockdown, compared with scrambled siRNA control. n = 3 and data are shown as z-scores. *p* values were calculated using a two-tailed unpaired *t*-test. * *p* < 0.05, *** *p* < 0.001, **** *p* < 0.0001.

We first looked in The Cancer Genome Atlas (TCGA) prostate adenocarcinoma (PRAD) Firehose cohort to compare *EDEM1*, *EDEM2* and *EDEM3* gene expression data in prostate adenocarcinoma tissue (n = 497) and normal prostate tissue (n = 52) [28]. *EDEM3* expression was the only significantly upregulated *EDEM* gene in prostate tumour tissue compared to normal tissue (*p* = 0.0009) (Figure 1A). Of interest, we observed large variation in *EDEM3* expression levels in this cohort.

We next sought to validate these findings in 4 other independent patient cohorts. We carried out a meta-analysis of 366 prostate tissue samples from four different studies [29,30,31,32] (Figure 1B). Our data show that *EDEM3* expression alone was consistently upregulated across our meta-analysis, being found, on average, in the top 2.25% of overexpressed genes in all four cohorts. *EDEM1* and *EDEM2* were found to be significantly overexpressed in only two of the four cohorts. Fold change data can be found in Supplemental Table S2 Our data suggest that across five independent clinical cohorts, *EDEM3* is the most highly expressed *EDEM* gene in prostate cancer and is the only *EDEM* paralog that is consistently upregulated in prostate cancer patient tissue.

To investigate the clinical implications of *EDEM* gene upregulation, we looked at *EDEM1/2/3* mRNA expression in relation to disease-free survival in 471 prostate cancer patients in the TGCA PRAD cohort [28] (Figure 1C–E). Survival analysis showed that high levels of *EDEM3* expression were significantly associated with a poorer disease-free survival rate (*p* ≤ 0.0001). Importantly, we found no significant association between EDEM3 mRNA expression and other clinical parameters which may affect survival (supplemental Figure S1). In contrast, neither *EDEM1* nor *EDEM2* gene expression correlated with disease-free survival in prostate cancer patients. These data show that *EDEM3* is the most clinically relevant *EDEM* paralog in prostate cancer.

We have previously shown *EDEM3* gene expression levels to be reduced in men post-androgen deprivation therapy. We next tested which *EDEM* genes are androgen responsive. We treated LNCaP (prostate adenocarcinoma cells) and RWPE-1 cells (normal prostatic epithelial cells) with 10 nM synthetic androgens (R1881) for 24 h and assessed gene expression of *EDEM1*, *EDEM2* and *EDEM3* (Figure 1F,G). *EDEM3* mRNA levels significantly increased following stimulation with synthetic androgens, compared with steroid-depleted controls (LNCaP *p* = 0.03; RWPE-1 *p* = 0.05). In contrast, there was no consistent expression change for either *EDEM1* or *EDEM2* in response to androgens. The regulation of *EDEM3* by androgens was further confirmed by depleting LNCAP cells of the AR (Figure 1H), which significantly decreased the levels of *EDEM3* (*p* = 0.02), whilst no statistically significant differences were detected for *EDEM1* or *EDEM2*.

### 2.2. Depletion of EDEM3 from Prostate Cancer Cells Increases Expression of ER Stress and Apoptosis-Associated Genes

**Figure 2:**
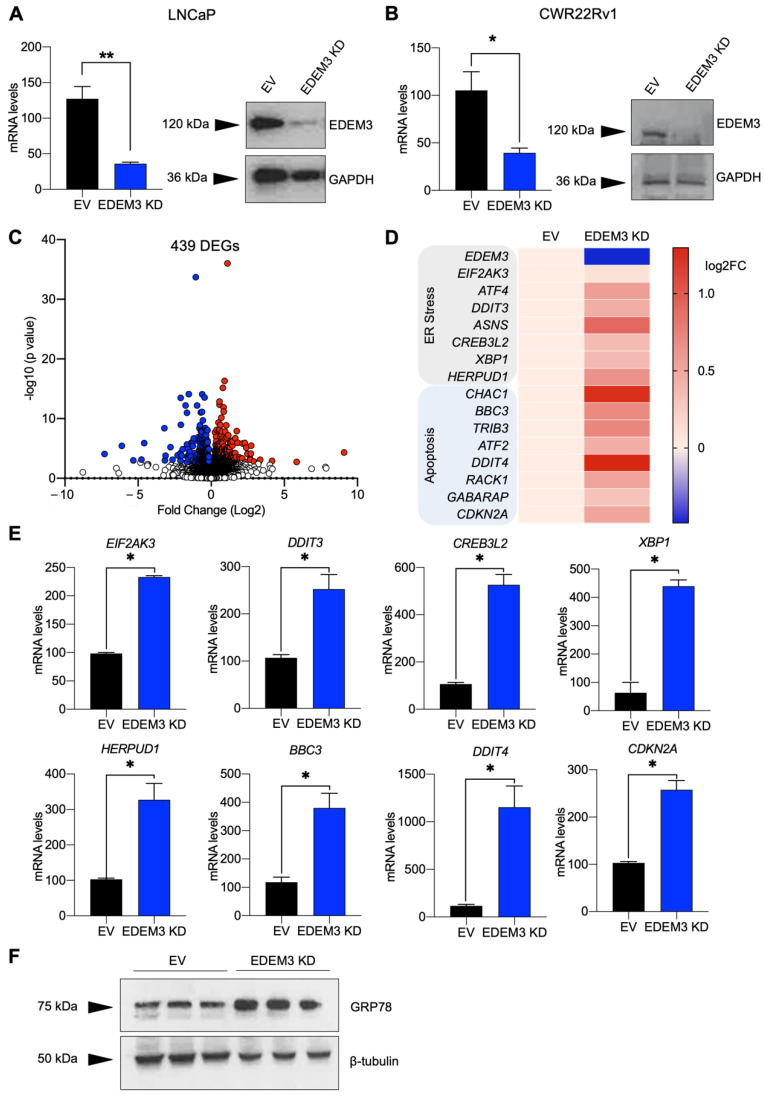
Depletion of EDEM3 from prostate cancer cells increases expression of ER stress and apoptosis-associated genes. (**A**,**B**) mRNA and protein levels of EDEM3 in LNCaP and CWR22Rv1 cells following shRNA-mediated *EDEM3* gene knockdown compared with empty vector control. mRNA levels shown as mean ± s.e.m. Protein levels detected by Western blotting. GAPDH was used as a loading control. (**C**) Volcano plot showing transcriptomic analysis of CWR22Rv1 cells following *EDEM3* gene knockdown. Significantly downregulated genes are shown in blue and significantly upregulated genes are shown in red. Significance was determined using an adjusted *p* value. * *p* < 0.05, ** *p* < 0.01. (**D**) Heatmap showing the log fold change in gene expression levels of ER stress and apoptosis-associated genes from RNA sequencing of CWR22Rv1 cells following *EDEM3* knockdown. (**E**) mRNA expression levels of ER stress-associated genes and pro-apoptotic genes following *EDEM3* knockdown in LNCaP cells. n = 3 and data are mean ± s.e.m. (**F**) Western blot analysis of GRP78 in LNCaP cells following EDEM3 gene knockdown.

Each of the above data indicate that *EDEM3* is the most clinically important *EDEM* paralog in prostate cancer. As such, we focussed on further understanding the role of *EDEM3* in prostate cancer biology. To interrogate the functional effect of *EDEM3* in prostate cancer, we next generated stable prostate cancer cell lines with knockdown of *EDEM3*. We achieved approximately 70–75% *EDEM3* gene knockdown in both LNCaP and CWR22Rv1 cells, and confirmed loss of EDEM3 at the protein level (Figure 2A,B).

We previously found that loss of EDEM3 in prostate cancer cells results in a significant decrease in cellular viability [4]. To understand why loss of *EDEM3* in prostate cancer cells reduces cell survival, we performed RNA sequencing of our CWR22Rv1 shRNA stable cell line. Differential gene expression analysis indicated that loss of *EDEM3* resulted in significant downregulation of 279 genes and significant upregulation of 391 genes based on an adjusted *p*-value of <0.05 (Figure 2C, Supplemental Table S3). Gene ontology (GO) analysis of differentially expressed genes revealed gene expression changes in processes linked to stress responses (Supplemental Table S4). These included significant changes in ER, UPR and apoptosis-associated processes, including ER-protein targeting (FDR < 0.0001) and ER-nucleus signalling (FDR ≤ 0.0001). Of interest, both the ‘unfolded protein response’ (FDR = 0.019) and the ‘PERK-mediated unfolded protein response’ (FDR = 0.002) were identified as altered processes following loss of *EDEM3* in prostate cancer cells. Our data also show changes in the cell-death-associated process, such as ‘regulation of cell death’ (FDR = 0.001) and ‘regulation of programmed cell death’ (FDR = 0.002).

Figure 2D shows a heatmap of gene expression changes which are associated with ER stress and apoptosis-associated GO terms. These differentially expressed genes include *XBP1* (adjusted *p* = 0.01) [33] and *ATF4* (adjusted *p* ≤ 0.0001) [34], *CHAC1* (adjusted *p* = 0.02) [35] and *BBC3* (adjusted *p* = 0.03) [36]. We next tested whether EDEM3 depletion in a second prostate cancer cell line would promote similar gene expression changes in UPR and apoptosis-associated genes. Using qPCR, we show that downregulation of *EDEM3* in LNCaP cells increases the expression of genes with roles in ER stress/UPR (*EIF2AK3*, *XBP1*, *DDIT3*, *CREB3L2* and *HERPUD1*) and apoptosis (*CDKN2A*, *ATF3*, *DDIT4* and *BBC3*) (Figure 2E). This is consistent with our previous study, where loss of EDEM3 reduced prostate cancer cell viability [4]. To confirm that loss of EDEM3 results in an increase in ER stress in prostate cancer cells, we measured expression of GRP78, a common marker of ER stress (Figure 2F). Western blot analysis of GRP78 confirmed that upon loss of EDEM3, LNCaP cells have higher levels of ER stress. These findings suggest that depletion of EDEM3 leads to an upregulation of ER stress and pro-apoptotic pathways.

### 2.3. EDEM3 Is Associated with ER Stress in Prostate Cancer Patients and Is Induced by ER Stressors

As loss of EDEM3 in our cell line models resulted in an increase in a UPR gene signature and the ER stress marker GRP78, we next investigated whether *EDEM3* is associated with ER stress in prostate cancer patients. Many pathological features of solid tumours, such as hypoxia and oxidative toxicity, can disrupt ER homeostasis, resulting in increased ER stress and activation of the unfolded protein response [37,38]. We tested whether levels of *EDEM3* expression correlate with UPR stress sensors in prostate cancer patient tissue. Strikingly, Spearman correlation analysis from the TCGA PRAD cohort (n = 493) [28] showed that *EDEM3* expression positively correlates with each of the UPR stress sensors; *ERN1* (IFE-1α), *EIF1AK3* (PERK) and *ATF6* (Figure 2A–C). This strongly indicates that *EDEM3* expression is associated with the UPR in prostate cancer clinical tissue.

**Figure 3:**
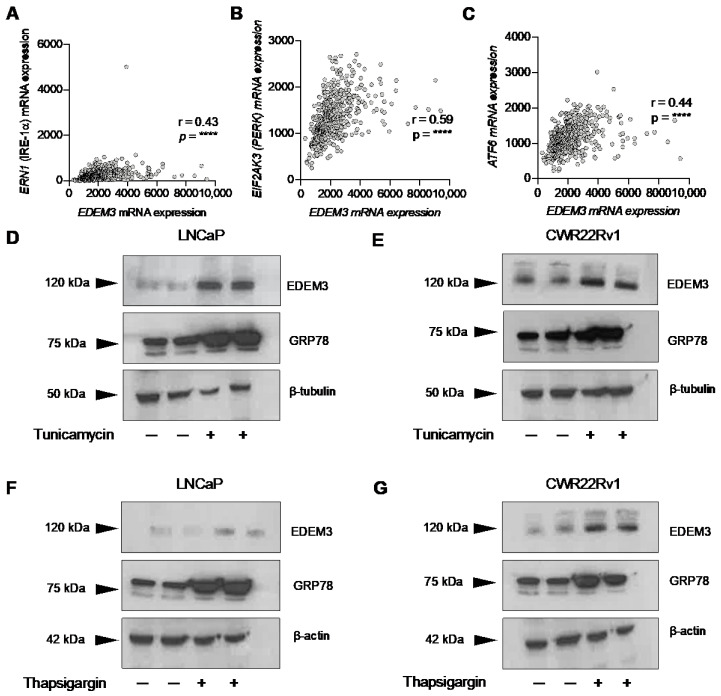
EDEM3 is associated with ER stress in prostate cancer patients and is ER-stress responsive. (**A**–**C**) Spearman correlation of EDEM3 gene expression with the three UPR stress sensors ERN1 (IRE-1α), EIF2AK3 (PERK) and ATF6. Gene expression values are a part of TCGA prostate adenocarcinoma (PRAD) cohort, accessed through CBioPortal. n = 493 [28]. mRNA expression values for EDEM3 were correlated with ERN1, EIF2AK3 and ATF6. *p* values were calculated using a two-tailed Spearman correlation with 95% confidence intervals. **** *p* < 0.0001. (**D**) Western blot analysis of EDEM3 and GRP78 in LNCaP cells in response to 2.5 µg/mL tunicamycin for 24 h. β–tubulin was used as a loading control. (**E**) Western blot analysis of EDEM3 and GRP78 in CWR22Rv1 cells in response to 2.5 µg/mL tunicamycin for 24 h. β–tubulin was used as a loading control. (**F**) Western blot analysis of EDEM3 and GRP78 in LNCaP cells in response to 100 nM thapsigargin for 24 h. β–actin was used as a loading control. (**G**) Western blot analysis of EDEM3 and GRP78 in CWR22Rv1 cells in response to 100 nM thapsigargin for 24 h. β–actin was used as a loading control.

Having already shown that loss of EDEM3 results in an induction of ER stress, we wanted to investigate whether EDEM3 itself is responsive to ER stress. We treated LNCaP and CWR22Rv1 cells with the ER stressing agents thapsigargin and tunicamycin for 24 h. Tunicamycin and thapsigargin are well established models of ER stress induction in vitro and act through two distinct pathways. Tunicamycin works by inhibiting *N*-glycosylation and thapsigargin by inhibiting calcium signalling. In the literature, varying culture conditions have been used, including different timepoints, inhibitor concentrations and serum levels. Several studies have used the inhibitors on prostate cancer cell lines, and we have used these studies to select our culture conditions for these experiments [39,40,41,42,43,44]. Following treatment with tunicamycin for 24 h, we detected levels of the ER stress marker GRP78 and EDEM3 using Western blotting. Our data confirm that treatment with tunicamycin induced ER stress in both LNCaP and CWR22Rv1 cells, as shown by an increase in GRP78 protein levels (Figure 3D,E). In response to tunicamycin mediated ER stress, we detected increased levels of EDEM3 in both CWR22Rv1 and LNCaP cells. Similarly, we confirmed that thapsigargin induced ER stress in our model, shown through an increase in GRP78 protein levels (Figure 3F,G). We also detected an increase in EDEM3 in response to thapsigargin exposure (Figure 3F,G). Taken together, these data show that *EDEM3* is correlated with ER stress markers in prostate cancer patients, and that it is upregulated in response to ER stress in prostate cancer cells.

### 2.4. EDEM3 Overexpression in Prostate Cells Protects Cells from ER Stressors

**Figure 4:**
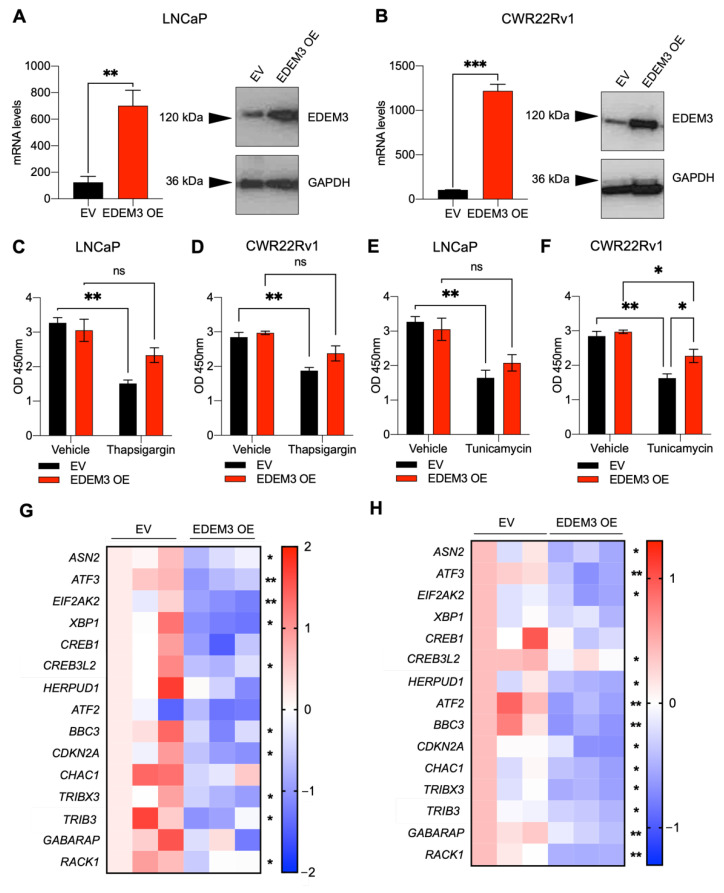
EDEM3 overexpression in prostate cells protects from ER stressors (**A**,**B**) mRNA and protein levels of EDEM3 in LNCaP and CWR22Rv1 cells following overexpression of EDEM3 compared with an empty vector control. Data are presented as mean ± s.e.m. Western blot analysis shown with GAPDH used as a loading control. (**C**–**F**) Cellular viability of LNCaP and CWR22Rv1 cells, with overexpression of EDEM3, treated with either vehicle (DMSO), 2.5 µg/mL tunicamycin or 100 nM thapsigargin for 24 h. Viability measured by WST-1 assay and shown as absorbance at O.D 450 nm. n = 3. Data are mean ± s.e.m. *p* values were calculated using two-tailed unpaired *t*-tests. Ns = not significant, * *p* < 0.05 and ** *p* < 0.01. (**G**) Heatmap showing mRNA levels of *ASN2, ATF3, EIF2AK3, XBP1, CREB1, DDIT3, HERPUD1, ATF2, BBC3, CDKN2A, CHAC1, TRIBX3, DDIT4, GABARAP* and *RACK1* in LNCaP cells following overexpression of EDEM3, compared with empty vector control. n = 3 and data are presented as z-scores. *p* values were calculated using two-tailed unpaired *t*-tests. * *p* < 0.05, ** *p* < 0.01. (**H**) Heatmap showing mRNA levels of *ASN2, ATF3, EIF2AK3, XBP1, CREB1, DDIT3, HERPUD1, ATF2, BBC3, CDKN2A, CHAC1, TRIBX3, DDIT4, GABARAP* and *RACK1* in CWR22Rv1 cells following overexpression of EDEM3 compared with empty vector control. n = 3 and data are presented as z-scores. *p* values were calculated using two-tailed unpaired *t*-tests. * *p* < 0.05, ** *p* < 0.01, *** *p* < 0.001.

As *EDEM3* is upregulated in prostate cancer tissue, we next created cell line models with overexpression of EDEM3. Both CWR22Rv1 and LNCaP cells were transfected with an EDEM3 expression vector or an empty vector control. In LNCaP cells, we achieved an approximately four-fold overexpression, and in CWR22Rv1, an approximately ten-fold overexpression as observed at the mRNA level by qPCR. EDEM3 protein overexpression was confirmed by Western blot (Figure 4A,B).

To understand the role of EDEM3 in the ER stress response in prostate cancer, we treated EDEM3 overexpressing cells with the ER stressing agents thapsigargin and tunicamycin for 24 h. In both LNCaP and CWR22Rv1 empty vector (EV) control cells, thapsigargin significantly decreased cellular viability (LNCaP *p* = 0.002; CWR22Rv1 *p* = 0.005). This effect was, however, ameliorated by EDEM3 overexpression, where, although there was a decrease in viability, it was no longer significant (LNCaP *p* = 0.16; CWR22Rv1 *p* = 0.13) (Figure 4C,D). In LNCaP cells treated with tunicamycin, we observed a statistically significant decrease in viability between EV control cells following treatment (Figure 4E). When comparing EDEM3 overexpressing cells, tunicamycin treatment reduced cellular viability, but this was not statistically significant (Figure 4E). In CWR22Rv1 cells treated with tunicamycin, although there was a significant reduction in viability in EDEM3 overexpressing cells following tunicamycin treatment, cellular viability was significantly higher with EDEM3 overexpression compared with EV control (*p* = 0.04) (Figure 4F). Although we only observed a significant increase in viability in tunicamycin-treated cells overexpression EDEM3 in our CWR22Rv1 cell line, EDEM3 overexpression in LNCaPs exhibited a smaller response, perhaps due to intrinsic differences in these two models.

Next, we used these models to investigate the effect of EDEM3 overexpression on ER stress and pro-apoptotic gene signatures. We used qPCR to monitor a panel of 15 genes linked to ER stress and apoptosis that were also previously identified as being differentially expressed following *EDEM*3 knockdown in our RNA sequencing screen. In LNCaP cells, EDEM3 overexpression resulted in a significant downregulation of several ER stress and apoptotic genes, including *EIF2AK3, XBP1, BBC3* and *RACK1* (Figure 4G). EDEM3 overexpression in CWR22Rv1 cells also led to a significant decrease in ER stress and apoptosis-related genes, including *EIF2AK3, HERPUD1, BBC3* and *CHAC1* (Figure 4H). This suggests that overexpression of EDEM3 in prostate cancer cells can lead to a reduction in ER stress and apoptosis-associated gene signatures.

### 2.5. Loss of EDEM3 Sensitises Prostate Cancer Cells to ER Stressors, and Its Upregulation Promotes Radio-Resistance

Having shown that in our model, EDEM3 overexpression can protect cancer cells from ER stress-inducing agents, we hypothesised that targeting EDEM3 may sensitise cancer cells to the ER stressors tunicamycin and thapsigargin.

**Figure 5:**
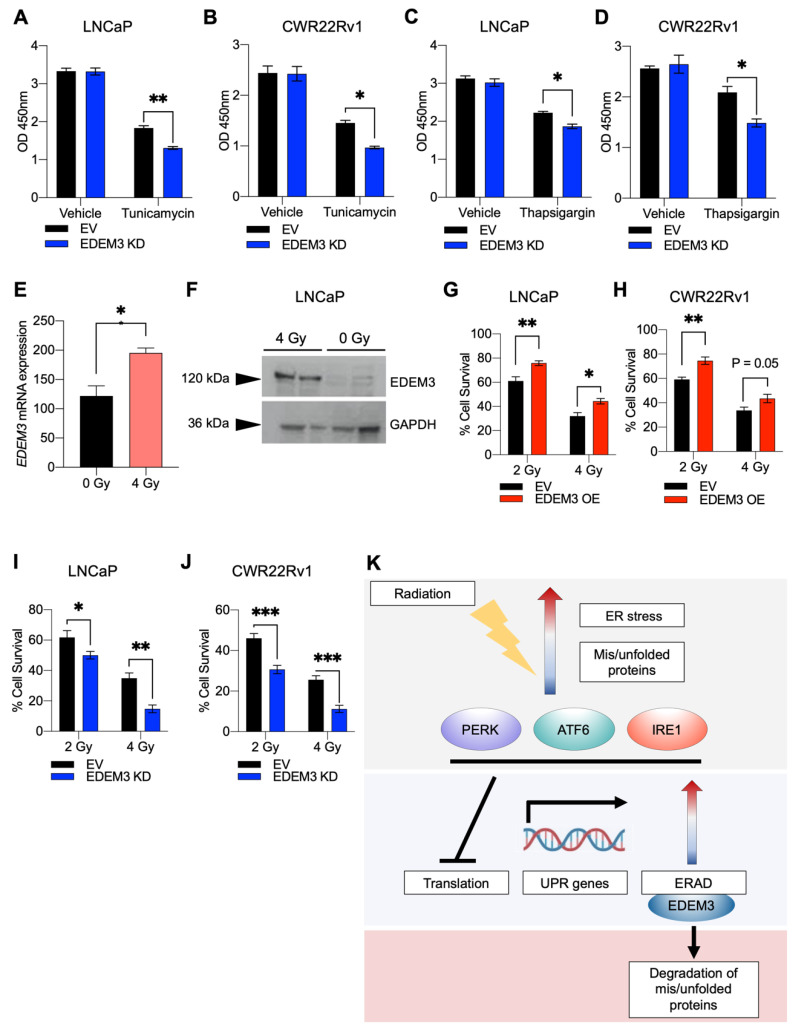
EDEM3 depletion in prostate cancer sensitises cells to ER stress and its upregulation promotes radio-resistance. (**A**–**D**) Cellular viability of LNCaP and CWR22Rv1 cells, with knockdown of EDEM3, treated with either vehicle (DMSO), 100 nM thapsigargin or 2.5 µg/mL tunicamycin, for 24 h. Viability measured by WST-1 assay and shown as absorbance at O.D 450 nm. n = 3. Data are mean ± s.e.m. *p* values were calculated using two-tailed unpaired *t*-tests. * *p* < 0.05 and ** *p* < 0.01 (**E**) mRNA levels of EDEM3 in LNCaP cells 48 h after exposure to 4 Gy radiation. Data are presented as mean ± s.e.m. *p* value calculated using a two-tailed unpaired *t*-test. * *p* < 0.05. (**F**) Western blot analysis of EDEM3 expression in LNCaP cells 48 h after exposure to 4 Gy radiation. GAPDH was used as a loading control. (**G**,**H**) Cell survival determined by clonogenic assay. LNCaP and CWR22Rv1 cells transfected with either an empty vector or EDEM3 overexpression vector were irradiated with either 2 or 4 Gy and left to form colonies for 14 days. At 14 days, any colony with more than 50 cells was counted. Cell survival was calculated by comparison with relative 0 Gy controls. n = 5. Data are mean ± s.e.m. *p* values were calculated using a two-way ANOVA. * *p* < 0.05, ** *p* < 0.01. (**I**,**J**) Cell survival determined by clonogenic assay. Stable EDEM3 knockdown and control LNCaP and CWR22Rv1 cells were irradiated with either 2 or 4 Gy and left to form colonies for 14 days. At 14 days, any colony with more than 50 cells was counted. Cell survival was calculated by comparison with relative 0 Gy controls. n = 5. Data are mean ± s.e.m. *p* values were calculated using a two-way ANOVA. * *p* < 0.05, ** *p* < 0.01 and *** *p* < 0.001. (**K**) Model of the role of EDEM3 in radio-resistance. Exposure to radiation induces ER stress and an accumulation of misfolded and unfolded proteins in cells. In response, the UPR is activated by three critical sensing proteins, PERK, ATF6 and IRE1. Activation of the UPR results in the inhibition of translation, transcription of UPR-associated genes and stimulation of ERAD. EDEM3, an important ERAD-associated enzyme, begins to trim mannose from misfolded glycoproteins to signal for degradation, aiding cell survival and protecting cells from radiation.

LNCaP and CWR22Rv1 cells with depleted EDEM3 were treated with tunicamycin (2.5 µg/mL) and thapsigargin (100 nM) (Figure 5A–D). In both LNCaP and CWR22Rv1 cells, treatment with either thapsigargin or tunicamycin resulted in a significant decrease in cellular viability, and this proved significantly more deleterious to cells with *EDEM3* gene knockdown (LNCaP thapsigargin *p* = 0.03; LNCaP tunicamycin *p =* 0.003; CWR22Rv1 thapsigargin *p* = 0.03; CWR22Rv1 tunicamycin *p* = 0.04). These data suggest that knockdown of *EDEM3* sensitises prostate cancer cells to ER stress.

Radiotherapy is often used in the clinical management of prostate cancer. DNA damage has been shown to activate members of the UPR in glioblastoma, breast cancer and colorectal cancers [45,46]. There is also broad evidence that overexpression of members of the UPR results in resistance to radiotherapy [47,48]. More recently, it has become clear that targeting the UPR may present an attractive opportunity to overcome radio-resistance [26,27]. As our data above showed that *EDEM3* expression levels are very closely associated with the UPR, we sought to test whether exposure to radiation could also induce *EDEM3* expression. To test this, we exposed LNCaP cells to radiation and analysed *EDEM3* gene and protein expression. A radiation dose of 4 Gy was selected based on previous studies with this cell line [49,50]. At 48 h post-irradiation, we observed a 60% increase in *EDEM3* gene expression and an increase in EDEM3 protein expression as detected by Western blot (Figure 5E,F).

Given the induction of EDEM3 expression in irradiated cells, we sought to establish the effect of EDEM3 overexpression on cellular viability in response to radiation exposure. We, therefore, performed clonogenic assays on both LNCaP and CWR22Rv1 cells overexpressing EDEM3 following exposure to either 2 or 4 Gy radiation (Figure 5G,H). An increase in cellular viability was observed in irradiated LNCaP and CWR22Rv1 cells with upregulated EDEM3 compared to control cells, suggesting that high levels of EDEM3 might confer radio-resistance to prostate cancer cells. As a proof-of-concept experiment to show that targeting the UPR via EDEM3 sensitises cells to radiotherapy, we subjected LNCaP and CWR22Rv1 cells to two doses (2 and 4 Gy) of radiation after *EDEM3* depletion (Figure 5I,J). Here, we observed a significant decrease in cell viability in irradiated cells with loss of EDEM3, with both LNCaP and CWR22Rv1 cell lines exhibiting a dramatic 50% reduction in cell viability with exposure to 4 Gy radiation (LNCaP *p* = 0.001; CWR22Rv1 *p* = 0.0003).

## 3. Discussion

EDEM3 is an EDEM protein paralog which plays a role in mannose trimming to promote the degradation of misfolded glycoproteins. Very few studies have been conducted examining the role of EDEM3 in human disease, and we were the first to implicate EDEM3 in human malignancies [4]. Here, using in vitro functional assays and transcriptomic analysis, we identify EDEM3 as an important ERAD- and UPR-associated gene, responsible for ensuring protection from ER stress in prostate cancer cells. Our data suggest that EDEM3 acts as a pro-survival factor in prostate cancer cells, and that upregulation of EDEM3 may protect cancer cells from ER stress and have implications for radio-resistance in prostate cancer cell lines.

The data presented in this study suggest that *EDEM3* is the EDEM gene which is preferentially upregulated in prostate cancer, showing consistent upregulation across 863 prostate cancer patients. Although it is consistently upregulated, we did observe large variations in its mRNA expression in patients, perhaps linked to the well documented heterogeneity of prostate tumours [51]. *EDEM1* and *EDEM2* were slightly increased in prostate cancer patients in two out of five cohorts, suggesting that they may play some role in ERAD in prostate cancer; however, their expression was in no way associated with disease-free progression. High *EDEM3* expression, however, was associated with a poor disease-free progression in patients. This is in line with our previous work suggesting that EDEM3 promotes cancer cell viability.

Transcriptomic analysis of EDEM3 knockdown cells, presented here, has corroborated a role for EDEM3 as a pro-survival factor, showing that following EDEM3 knockdown, expression levels of pro-apoptotic and UPR genes increase. Whilst we see an increase in transcription of pro-apoptotic genes, we did not observe a decrease in viability in cells not exposed to a stressing agent. As much of the literature around EDEM3 focuses on its role as an ERAD-associated enzyme, cells may need to be in a stressed condition before a loss of EDEM3 translates to a decrease in viability. In 2006, Hirao et al. established a role for EDEM3 in the quality control process, responsible for recognising and correctly disposing of misfolded glycoproteins, with a specific α1,2-mannosidase activity, distinct from the mannose trimming capabilities exhibited by other EDEM paralogs [22]. Protein mis-folding and ER stress are biproducts of stress-inducing conditions in tumours such as hypoxia, nutrient deficits, high metabolic demand and oxidative stress [52,53,54]. The UPR and ERAD are important pro-survival pathways which tumours exploit to balance ER homeostasis, ensuring their continued survival and growth [55]. Although EDEM3 had previously been identified as a vital ERAD-associated protein, its role in the UPR and ERAD in cancer had yet to be explored. Our RNA-sequencing analysis supports the idea that EDEM3 is an important member of the ERAD family, identifying ER stress and the UPR as factors affecting cell survival in EDEM3 knockdown cells. Importantly, we have shown EDEM3 to be associated with ER stress using multiple stressors (tunicamycin, thapsigargin and irradiation), suggesting that independent of the method of ER stress induction, EDEM3 is linked to ERAD.

Targeting proteins involved in the UPR and ERAD have previously been shown to sensitise cells to ER stressors [56,57,58]. As we have shown EDEM3 to be a cyto-protective against ER stressors, we suggest that targeting of EDEM3, shown here through gene knockdown, may be an effective strategy to sensitise cancer cells to ER stress-inducing agents. Taking this further, many are now looking at the UPR and ERAD components as key mediators of resistance to common therapies such as radiotherapy and chemotherapy [55,59,60]. Our in vitro data suggest that, in these models, high levels of EDEM3, as well as promoting cell survival, may promote resistance to radiation therapy. Although factors which modulate radiosensitivity have been identified, such as DNA damage and reactive oxygen specifies, the mechanisms of acquired radio-resistance in prostate cancer are still poorly understood [61,62]. Further studies, including in vivo analyses, may help to decipher specific mechanisms of radio-resistance. Future work may investigate a clinical role for EDEM3 as a therapeutic target in combination with radiotherapy for prostate cancer. Here, we present proof-of-concept data to show for the first time that targeting EDEM3 may provide an opportunity to reduce levels of radio-resistance, and that the role of EDEM3 as a therapeutic target for prostate cancer warrants further investigation.

## 4. Materials and Methods

### 4.1. Cell Culture and Generation of Stable Cell Lines

Cell culture and the cell lines used were as described previously [4]. LNCaP and CWR22Rv1 cell lines were maintained in RPMI-1640 medium, supplemented with 10% foetal bovine serum and 1% penicillin streptomycin. RWPE-1 cells were maintained in keratinocyte serum-free media (gibco) supplemented with 1% penicillin streptomycin, 0.05 mg/mL bovine pituitary extract and 5 ng/mL epidermal growth factor.

For the stable knockdown of EDEM3, control and EDEM3 shRNA lentiviral particles were purchased from Santa Cruz (sc108080 and sc-78683-V). Transductions were carried out according to the manufacturer’s protocol and a multiplicity of infection (MOI) of 5 was used for both shControl and shEDEM3. Successfully transduced cells were selected using 5 µg/mL puromycin.

To generate cell lines with stable overexpression of EDEM3, an EDEM3 cDNA ORF clone in a pcDNA3.1-C-(k)DYK expression vector was purchased from Genscript (clone number: OHu04410) and transfected into 0.2 × 10^6^ LNCaP or CWR22Rv1 cells in a 6-well dish using lipofectamine 3000 according to the manufacturer’s protocol. A pcDNA3.1-C-(k)DYK empty vector was used as the empty vector control. Successfully transfected cells were selected for using 5 µg/mL puromycin.

### 4.2. Cell Treatments

Androgen stimulation experiments were carried out as described previously [63]. For experiments in which LNCaP and RWPE-1 cells were stimulated with androgens, cells were cultured in steroid deplete media (media supplemented with dextran charcoal-stripped foetal bovine serum) for 72 h, at which point 10 nM synthetic androgen analogue (R18811) (Perkin–Elmer, NLP005005MG) was added for 24 h. The RNA samples used for this study have been previously validated and published [63].

Tunicamycin was purchased from Sigma (T7765), resuspended in DMSO and used at a concentration of 2.5 µg/mL for all experiments. Thapsigargin was purchased from Sigma (T9033) resuspended in DMSO and used at a concentration of 100 nM for all experiments. Cells were stimulated with ER stressors for 24 h before conducting cellular viability assays. Control cells were treated with DMSO as a vehicle control.

### 4.3. RNA Sequencing

CWR22Rv1 cells with stable *EDEM3* knockdown and mock-depleted cells treated with control shRNA lentivirus were used for RNA sequencing. RNA was extracted using the Qiagen RNAeasy kit (74104) according to manufacturer’s protocol. The RNA sequencing was performed at Newcastle University Genomics Core Facility using TruSeq Stranded mRNA Sequencing NextSeq High-Output to obtain 2 × 75 bp reads. Quality control of reads was performed using FastQC. Reads were mapped to the hg38 transcriptome using Salmon. Differential gene expression analysis was performed using DESeq2. Data were further analysed using the gene ontology resource (http://geneontology.org) (accessed on 3 June 2020) [64,65].

### 4.4. Quantitative PCR (qPCR)

Cells were harvested and RNA extracted using Tri-reagent (Invitrogen, Waltham, MA, USA, 15596-026) according to the manufacturers protocol. CDNA synthesis was performed on 500 ng of RNA using the Superscript VILO cDNA synthesis kit (Invitrogen, 11754-050). Quantitative PCR (qPCR) was performed in triplicate using SYBR^®^ Green PCR Master Mix (Invitrogen, 4309155) and the QuantStudio 7 Flex Real-Time PCR System (Life Technologies, Carlsbad, CA, USA). Gene expression values were normalised to the average of three housekeeping genes: GAPDH, β–tubulin and actin. Primer sequences available in Supplemental Table S1.

### 4.5. Clonogenic Assays

Cells were irradiated at room temperature in a T75 flask at a dose of either 2 or 4 Gy per minute. Cells were then trypsinised and detached from the flask and then plated at an appropriate density in 100 mm dishes and maintained for 14 days until colonies of more than 50 cells/colony had formed. Cells were then fixed at room temperature in 10% formalin for 15 min and stained with 0.5% crystal violet for 10 min at room temperature. The number of colonies containing >50 cells were then counted as representative of cell survival. Cell survival was calculated as a percentage compared with non-irradiated controls.

### 4.6. Statistical Analyses

All statistical analyses were performed using GraphPad Prisms 8 (GraphPad Software, Inc., San Diego, CA, USA). Data are presented as the mean of three independent samples ± standard error of the mean (SEM). Statistical significance is indicated as * *p* < 0.05, ** *p* < 0.01, *** *p* < 0.001 and **** *p* < 0.0001.

## Supplementary Materials

The following supporting information can be downloaded at: https://www.mdpi.com/article/10.3390/ijms23158184/s1.
